# Karl Pribram, the James Arthur Lectures, and What Makes Us Human

**DOI:** 10.1186/1747-5333-1-15

**Published:** 2006-11-29

**Authors:** Ian Tattersall

**Affiliations:** 1 Division of Anthropology, American Museum of Natural History, New York NY 10024, USA

## Abstract

**Background:**

The annual James Arthur lecture series on the Evolution of the Human Brain was inaugurated at the American Museum of Natural History in 1932, through a bequest from a successful manufacturer with a particular interest in mechanisms. Karl Pribram's thirty-ninth lecture of the series, delivered in 1970, was a seminal event that heralded much of the research agenda, since pursued by representatives of diverse disciplines, that touches on the evolution of human uniqueness.

**Discussion:**

In his James Arthur lecture Pribram raised questions about the coding of information in the brain and about the complex association between language, symbol, and the unique human cognitive system. These questions are as pertinent today as in 1970. The emergence of modern human symbolic cognition is often viewed as a gradual, incremental process, governed by inexorable natural selection and propelled by the apparent advantages of increasing intelligence. However, there are numerous theoretical considerations that render such a scenario implausible, and an examination of the pattern of acquisition of behavioral and anatomical novelties in human evolution indicates that, throughout, major change was both sporadic and rare. What is more, modern bony anatomy and brain size were apparently both achieved well before we have any evidence for symbolic behavior patterns. This suggests that the biological substrate underlying the symbolic thought that is so distinctive of *Homo sapiens *today was *exaptively *achieved, long before its potential was actually put to use. In which case we need to look for the agent, perforce a cultural one, that stimulated the adoption of symbolic thought patterns. That stimulus may well have been the spontaneous invention of articulate language.

## Background: James Arthur and the James Arthur lecture series

Born in Ireland in 1842 and raised in Glasgow, Scotland, James Arthur immigrated to New York City in 1871. Skilled in mechanics and gear-cutting, he established a profitable career in the manufacture and repair of machinery of diverse types, in the course of which he founded a number of successful businesses and received patents on a variety of mechanical devices. Throughout his life he maintained a particular interest in horology, the science of measuring time. Early in the twentieth century James Arthur began an association with the American Museum of Natural History, as a result of which his interest in time-keeping expanded to evolutionary time and his fascination with mechanisms broadened to that most intricate and delicate mechanism of all, the human brain. His curiosity about how human beings came to be the behaviorally extraordinary creatures they are led ultimately to a bequest to the American Museum that, following his death in 1930, permitted the establishment of the annual distinguished lecture series, the James Arthur Lectures on the Evolution of the Human Brain. The first lecture in the James Arthur series was delivered on March 15, 1932, by Frederick Tilney, a distinguished neurologist who was Chairman of the Department of Neurology at Columbia University Medical Center from 1920 to 1935, and who is perhaps best remembered for his bold prediction that "We will by conscious command evolve cerebral centers which will permit us to use powers that we now are not even capable of imagining." In retrospect, it is evident that Dr Tilney's chosen title, *The Brain in Relation to Behavior*, accurately predicted the wide-ranging scope that has characterized lectures in the James Arthur series ever since.

Despite their diversity, however, few James Arthur presentations have ranged as widely as Karl Pribram's thirty-ninth lecture in the series, *What Makes Man Human*, delivered on April 23, 1970. In what must have seemed like a very short hour (alas, I was not there to witness it: Pribram's was almost the last James Arthur Lecture to be given before I joined the American Museum, and the speaker selection committee, in 1971), Pribram's seminal ruminations ran the gamut from the nature of information coding and the brain as machine, to the algorithms on which neural motor mechanisms function, through meaning and symbol and symbol-formation and finally, via a detour into chimpanzee communication, back to language and the symbol-mediated construction of meaning. All serving as prologue to his brief but pregnant answer to the question posed in his title: "man's brain is different in that it makes imperative the productive use of linguistic signs symbolically and linguistic symbols significantly" [ref. [1]](see page 31). In one short talk Pribram elegantly, compactly and comprehensively articulated an agenda that has underpinned much subsequent research on the compelling but infuriatingly elusive question of what it is that makes people human.

## Discussion

Perhaps predictably, the "what makes us human" question has in recent years given rise set many researchers to the introspective and often obsessive task of compiling laundry-lists of human behavioral uniquenesses, as exemplified by Donald Brown's book *Human Universals *[2]. But while this is superficially an attractive approach to understanding exactly what it is that makes us *different *from all other organisms, it really gets us nowhere. Partly this is because such lists can never be exhaustive. There is always something else to add: only we play bingo, only we grow bonzai plants, or wear lipstick, or whatever. And who is to say which activity in this potentially infinite list is the critical one, or even if any particular list includes that crucial activity – should one exist at all. Mainly, though, this approach to the question of what makes us different leads us up a blind alley because, as Karl Pribram evidently knew back in 1970, all of our peculiar activities stem from the more generalized underlying faculty that, in his fifty-fourth James Arthur Lecture of 1985, Alexander Marshack dubbed "the human capacity" [3]. Everything in our lists is effect, not cause. Still, many evolutionary psychologists have grasped at such listings of "key" human behavioral attributes as sources for the construction of a huge variety of just-so stories depicting the bizarre or merely unusual behaviors of modern human beings as lingering and no longer functional adaptations to conditions that reigned in the ancestral "environment of evolutionary adaptedness" [e.g. refs. [4,5]]. In turn, this mindset has given rise to notions of a "modular" evolution of the human cognitive capacity, whereby natural selection has led step-by-step to the gradual emergence of the modern human mind via the accretion of new and increasingly specialized aspects of intelligence [4,6]. Following Tooby and Cosmides (in ref. [4]), Mithen [6] has likened the human mind and its many proposed modules to a Swiss Army knife with many different blades, each module likely posessing "its own specific form of memory and reasoning process" [6](see page 43).

To the reductionist human mind this mechanistic explanation of human intelligence, like the stories that result from it, is undeniably attractive. We are a storytelling species, and the notion of natural selection as the inexorable propulsive force behind our own most distinctive attribute makes a compelling story. Yet a moment's thought is enough to indicate that evolutionary reality must be much more nuanced than this narrative suggests. Take the concept of natural selection itself. The basic notion here is that "fitter," or better adapted, individuals will outreproduce the less fit, and thus that their heritable advantages will become commoner in the consequently evolving population. On the face of it, in any species in which more offspring are produced than survive to reproduce themselves this process looks not merely plausible, but inevitable. Yet this reasoning really only works when we think of beneficial characteristics as existing and evolving in isolation from others. The reality is that each organism is an integrated whole, made up of huge numbers of mostly polygenic traits that are typically specified by pleiotropic genes that may be variably expressed. And natural selection can by its very nature only vote up or down on the reproductive success of the whole individual. It cannot single out individual features, still less particular genes, to favor or to eliminate. Yet this is the assumption on which the modular notion of brain/mind evolution depends. In other words, it is profoundly misleading to proceed as if each attribute under consideration somehow exists separately from the entire organism in which it is embedded, and as if each attribute has had an evolutionary history independent not only of its possessors, but also of the other traits that together make up the functioning whole.

Of course, some features of the organism may well make an absolutely critical contribution to reproductive success, and thus be individually subject to strong natural selection. But very probably such attributes are limited largely to those that are central to the reproductive process itself. It is, for example, very likely to be no accident that testis size is consistently greatest in those primate species with polygamous mating systems. Chimpanzee males compete vigorously for females, and it is very highly plausible that their remarkably large testes result directly from a history of sperm competition, a point forcefully made by Paul Harvey in his 1990 James Arthur Lecture, *Comparing Brains*. Gorilla males, on the other hand, may have smaller testes because an excess of metabolically expensive reproductive tissue is unnecessary where single males monopolize groups of females for extended periods of time.

Still, most animal tissues and activities are not devoted to reproduction per se but rather to economic ends, and neither affect nor reflect reproduction except in indirect and complex ways. If an individual is not economically successful it is highly unlikely to be reproductively successful, and in this sense it is possible to contend that reproductive structures and activities are little more than a veneer imposed on the basic economic machinery. Natural selection of the kind that promotes population change rather than population stability probably acts typically to propagate a very large and diverse subgroup of reproductively superior individuals whose economic performance is not necessarily any more than simply adequate. If individuals are the target of natural selection, and populations are the effective evolutionary units, then evolution cannot in itself be a process of optimization of characters; and what succeeds most of the time is merely what works. Reproductive success often will not involve being the best, but simply being good enough, in whatever respects happen to be important in prevailing circumstances. Local diversification within any species is a critically important evolutionary phenomenon, and in conjunction with speciation it provides a crucial link between microevolutionary and macroevolutionary processes; but most of the time, rather than being directional, it seems to work within what (to misappropriate a term from Niles Eldredge, ref. [7]) might be termed a "sloshing bucket" principle, whereby genetic and phenotypic frequencies move back and forth within the containing wall of the species, the occasional overflow being lost. The economic competition among entities that appears to be of more routine evolutionary importance is among species as wholes; and it is, after all, of very little use in the long run to be the best adapted individual of your species – whatever that may in practice mean – if your entire species is being outcompeted into extinction.

There are, then, many theoretical as well as practical reasons to reject the notion that human cognitive evolution inevitably consisted of a sort of mental fine-tuning via the continual accretion or modification of discrete modules, each with its own neural circuitry. And if we are as a result to abandon the notion of a human capacity that emerged in a series of tiny discrete steps, we find ourselves essentially back where Karl Pribram found himself in 1970, looking for the origin of a much less specific neural competence that underpins the entire range of our remarkable cognitive skills. The question then becomes whether this more general-purpose capacity arose through some sort of gradually-acting feedback mechanism that mediated a process of gradual refinement, or whether it represents a true innovation, a quantitative rather than a qualitative shift in cognitive functioning. Sheer brain size – which we can determine, if only spottily, from fossil endocasts [8] – turns out to be an inadequate measure of cognitive quality [9,10]; so to evaluate this question we have to look at the patterns of innovation that we see in the archaeological record. This record, the archive of hominid behavioral evolution as reflected in the material products of the hominids concerned, was inaugurated by the invention of stone tools some 2.6 million years ago.

The earliest stone toolmakers simply sought to obtain a sharp cutting edge by striking one fist-sized cobble with another. What the resulting sharp flake actually looked like was not a consideration. Still, simple cutting flakes were clearly a highly advantagous innovation, which saw no major improvement for a million years. But at about 1.6 myr ago toolmakers began to make "handaxes." These were implements of a standardized and symmetrical shape that evidently corresponded to a "mental template" that existed in the minds of the toolmakers before work began. And while one might intuitively expect that, as presumptive evidence of a significant cognitive advance, this radical innovation might have proved to be associated with a new kind of hominid, it was in fact accomplished by a hominid (albeit of a new kind) that had already been around for several hundred thousand years *before *this innovation was made. Appearing on the scene at a little under two million years ago, this hominid species, most commonly known as *Homo ergaster*, contained the first hominids that were of essentially modern body size and build, and that were thus radically different anatomically from their predecessors. Yet its initial representatives continued to display technological behaviors that were effectively identical to those that their much more archaically-proportioned antecedents had been wielding for half a million years and more. Still, even with brains a little bigger than those of their diminutive precursors – though still at best not much more than half the size of ours today – members of *Homo ergaster *were the first hominids to be truly emancipated from the forest edge and woodland habitats to which their precursors were largely confined. And, still wielding only crude stone tools, they rapidly spread far beyond Africa.

Following the invention of the handaxe, there is once again a long wait for the next major technological innovation, wherein a stone "core" was carefully shaped until a single blow would detach a more or less finished tool. This, too, surely signifies another notching-up in hominid cognitive complexity. And again, this invention came long after a new kind of hominid had shown up in the fossil record, at about 600 thousand years (600 kyr) ago in Africa and shortly thereafter in Eurasia. It was hominids of this new species, *Homo heidelbergensis*, that some 200 kyr later apparently introduced such important novelties as the building of shelters and the regular domestication of fire in hearths. There is, however, nothing in the archaeological record of these hominids that convincingly suggests symbolic activities. As ever, the pattern is of a total disconnect between the arrival of new kinds of hominid and the appearance of new kinds of stone tools. And significant change, whether behavioral or anatomical, continued to be not only sporadic, but rare.

Perhaps the most accomplished practitioners of prepared-core toolmaking were the members of *Homo neanderthalensis*. By virtue of a large fossil and archaeological record it is this species, which flourished in Europe and western Asia following about 200 kyr ago, that provides us with the best yardstick by which to judge the uniqueness of our own species, *Homo sapiens*. But while the Neanderthals had brains as large as ours are, invented the burial of the dead, and probably took care of disadvantaged members of society, they left little evidence to suggest that they possessed symbolic consciousness, the quality that Pribram was to single out as the key to human cognitive uniqueness, and to which numerous subsequent authors have also pointed (see ref. [11]). And it was the Neanderthals who, beginning some 40 kyr ago (and paralleled by similar but independent events that apparently occurred in eastern Asia at about the same time), were somehow driven to extinction in not much more than ten millennia by arriving *Homo sapiens *whose existences were very clearly drenched in symbol. These early European *Homo sapiens*, known as the Cro-Magnons, created astonishing art on the walls of caves. They carved exquisite figurines. They decorated everyday objects, and made notations on plaques of bone. They played music on bone flutes, and without question sang and danced as well. In short, they were *us*. And this complex material record they left behind is distinguished most notably from those of their non-African predecessors and contemporaries by its clear indications of a symbol-based mode of cognition. Like us, the Cro-Magnons lived not in the world as presented to them by Nature, but in a world they reconstucted in their own minds and were thus able to manipulate.

Still, the Cro-Magnons were not the first creatures who *looked *just like us. As suggested by such fossils as the Herto 1 and Omo Kibish 2 crania, the highly characteristic bony anatomy of modern *Homo sapiens *may have had its roots in Africa perhaps as long as 160–200 kyr ago [12,13], long before we find the possible earliest intimations of symbolic behaviors in that continent at about 100-80 kyr ago, at such sites as Klasies River Mouth and Blombos Cave [14]. Similarly, while anatomically modern *Homo sapiens *shows up for the first time in the Levant at a little under 100 kyr ago, these hominids made stone tools virtually indistinguishable from those made by the Neanderthals who continued to persist alongside or in alternation with them. The final disappearance of the Neanderthals from the Levant came only following the appearance there (apparently an indigenous development) of stone tools equivalent to those the Cro-Magnons brought with them fully formed into Europe. This appears to suggest that neither hominid species had an overall competitive advantage as long as the behaviors of both could be described as the most sophisticated extrapolations yet of the trends toward increasing brain size and cognitive complexity – in both lineages – that had preceded them. But once *Homo sapiens *began to behave in a "modern" fashion, the Neanderthals were faced with an entirely unanticipated phenomenon. And with its advent the rules of the game changed entirely. With its acquisition of symbolic consciousness *Homo sapiens *became an irresistible force in Nature, intolerant of competition from close relatives and possessed of the ability to indulge that intolerance.

There is nothing in the record just summarized to suggest that the acquisition of modern symbolic consciousness marked the culmination of a gradual trend through time, under the beneficent supervision of natural selection. Certainly, the acquisition of modern human cognition was *based *on what had gone before, and could not have happened without it. But the event itself marked a qualitative leap, rather than a small final accretionary step in an inexorable process of refinement. Some studies have pointed to hints in the earlier record of certain aspects of behavior that we commonly associate with modern humans [15]; but very likely straws in the wind such as blade and point production, long-distance exchange of materials, pigment grinding, and shellfishing (all of them essentially economic activities) merely point to a complex form of cognition that was nonetheless not symbolic. In this context it is important not to be misled by the undoubted steady increase in the average size of hominid brains over the past two million years. For while the minimalist taxonomy preferred by most paleoanthropologists has made it possible for many to assume a linear trend in this respect, what we are more probably seeing are the averaged effects of the preferential survival of larger-brained *Homo *species. Nonetheless, there was undeniably something about members of the genus *Homo*, or even of the family Hominidae, that predisposed them to the metabolically expensive process of brain enlargement; and knowing what that something was will certainly be crucial to fully understanding how modern human cognition was acquired.

All this notwithstanding, large brains by themselves are clearly not enough to assure symbolic consciousness. Neanderthals had brains of modern human size – indeed, with an endocranial capacity of 1740 ml, one Neanderthal had the largest fossil hominid brain ever reported – but, cognitively sophisticated as they doubtless were, they failed to leave convincing evidence of symbolic behaviors, certainly in "pre-contact" times. Perhaps more remarkably, this was also true for the first anatomically modern humans. The earliest potential *Homo sapiens *fossils in Africa are associated with remarkably unsophisticated stone artifacts, and the early moderns of the Levant some 90 kyr ago made stone tools just like those of the Neanderthals, with little if any sign of symbolic behaviors. So, at least as far as can be told from an admittedly indirect and incomplete record, there was a very considerable time-lag between the acquisition of modern anatomy and the expression of modern behavior patterns, putatively as long ago as 75 kyr or more in Africa and most dramatically expressed in Europe following about 40 kyr ago. It has proposed that an enabling genetic change, the effects of which were limited to brain activity, may have occurred and spread within *Homo sapiens *in the period following about 50 kyr ago. However, the spread would have had to have been very rapid indeed, and there is no independent suggestion of massive population replacement. More likely the structural neural capacity that underwrites the faculty for symbolic thought emerged with the substantial biological reorganization that accompanied the emergence of *Homo sapiens *as a readily recognizable anatomical entity, at some point over 150 kyr ago. That potential then lay long undiscovered, until it was released by what must have been a cultural rather than a biological stimulus.

Karl Pribram [1] identified a close relationship between symbolic cognition and language. He saw linguistic sentences as codes constructed by predication, a symbolic process that involved placing "linguistic signs into a context dependent frame" [1](see page 26). Predication is in turn "a statement of belief," which may exist in various degrees of certainty. And it is "this process of making statements of certainty which is unique to man" [1](see page 27). For Pribram, what made the cognition of human beings *different *from any other known is the "reciprocal interaction between sign and symbol." Pribram proposed this linkage in the context of asking just what it is about the human brain that makes symbolic thought possible (for which his answer was the "massive cortico-cortical connectivity" noted by another James Arthur lecturer, Norman Geschwind [16], added to the ubiquity of cortico-subcortical connections). But in the context of the emergence of symbolic cognition (as opposed to its substrate, which must obviously already have been present), it is equally notable that the invention of language (or at least of those elements of language dependent on symbolism) is the most plausible releasing factor. Language itself cannot have appeared entirely de novo. It clearly descended in some way from sophisticated earlier forms of vocal communication, the details of which are actively debated [e.g. [17,18]]. But articulate language equally clearly represents a qualitative leap away from any other form of such communication that we know of. And it should be noted that, by the time that we have any good inferential evidence for language use, the vocal apparatus necessary for speech production was already in place; for, if there is any correspondence at all between this activity and the production of symbolic artifacts and signs of complex manipulation of the environment, the transition to language evidently took place subsequent to the appearance of anatomically modern *Homo sapiens*. Language is the ultimate symbolic activity, involving as it does the creation of intangible symbols and their recombination in the mind to allow the asking of questions such as "what if?" And (unless its most important role is as an interior conduit to thought, rather than as a means of communication) language is in addition a communal property, which makes it more credible in the role of releaser than other suggested facilitators of symbolic thought such as theory of mind [e.g. [19]], which are internalized.

But whatever it may have been that ushered in the beginnings of symbolic thought in a *Homo sapiens *that had, like all other organisms, lived until then in a concrete external world rather than in one which it constantly mentally remade, it is clear that symbolic thought itself cannot have been propelled into existence by natural selection. Indeed, natural selection is *not *a creative force in the sense that it stimulates novelty; it can only act on variations that have come into existence spontaneously, and independently of context. This is not to deny the potential power of natural selection to mold populations under certain circumstances; but there is an argument to be made that all useful novelties – indeed, any novelties at all – have to arise not as *adaptations *but as *exaptations*: in the broad sense, as features not acquired in the context of the function to which they will eventually be put. No surprise here: everyone can agree that nothing arises *for *anything, and that selection can only work with what is already there. Moreover, once arisen, novelties may persist in populations for no better reason than that they do not get in the way, whether or not new uses for them might ultimately be discovered. This places the origin of our vaunted human cognitive capacities squarely in the arena of emergence: the chance acquisition in a compound organ system of a level of complexity that is greater than the sum of its individual components. This is a notion that is entirely in harmony with Karl Pribram's vision of 1970; and that perhaps, in removing evolutionary fine-tuning from the equation, makes it easier to understand his coda [1](seepage 32), a quote from A. J. Heschel: "to be human is to be a problem" [20](see page 105).

## Competing interests

The author(s) declare that they have no competing interests.
